# Application of Weighted Gene Coexpression Network Analysis to Identify Key Modules and Hub Genes in Systemic Juvenile Idiopathic Arthritis

**DOI:** 10.1155/2021/9957569

**Published:** 2021-08-13

**Authors:** Mi Zhou, Ruru Guo, Yong-Fei Wang, Wanling Yang, Rongxiu Li, Liangjing Lu

**Affiliations:** ^1^Department of Rheumatology, Ren Ji Hospital, School of Medicine, Shanghai Jiao Tong University, 145 Middle of Shandong Road, Huangpu District, Shanghai 200001, China; ^2^Department of Paediatrics and Adolescent Medicine, Li Ka Shing Faculty of Medicine, The University of Hong Kong, Pok Fu Lam, Hong Kong; ^3^State Key Laboratory of Microbial Metabolism and School of Life Sciences and Biotechnology, Shanghai Jiao Tong University, 800 Dong Chuan Road, Shanghai 200240, China

## Abstract

Systemic juvenile idiopathic arthritis (sJIA) is a severe autoinflammatory disorder with a still not clearly defined molecular mechanism. To better understand the disease, we used scattered datasets from public domains and performed a weighted gene coexpression network analysis (WGCNA) to identify key modules and hub genes underlying sJIA pathogenesis. Two gene expression datasets, GSE7753 and GSE13501, were used to construct the WGCNA. Gene Ontology (GO) and Kyoto Encyclopedia of Genes and Genomes (KEGG) enrichment analyses were applied to the genes and hub genes in the sJIA modules. Cytoscape was used to screen and visualize the hub genes. We further compared the hub genes with the genome-wide association study (GWAS) genes and used a consensus WGCNA to verify that our conclusions were conservative and reproducible across multiple independent datasets. A total of 5,414 genes were obtained for WGCNA, from which highly correlated genes were divided into 17 modules. The red module demonstrated the highest correlation with the sJIA module (*r* = 0.8, *p* = 3*e*^−29^), whereas the green-yellow module was found to be closely related to the non-sJIA module (*r* = 0.62, *p* = 1*e*^−14^). Functional enrichment analysis demonstrated that the red module was mostly enriched in the activation of immune responses, infection, nucleosomes, and erythrocytes, and the green-yellow module was mostly enriched in immune responses and inflammation. Additionally, the hub genes in the red module were highly enriched in erythrocyte differentiation, including *ALAS2*, *AHSP*, *TRIM10*, *TRIM58*, and *KLF1*. The hub genes from the green-yellow module were mainly associated with immune responses, as exemplified by the genes KLRB1, KLRF1, CD160, and KIRs. We identified sJIA-related modules and several hub genes that might be associated with the development of sJIA. Particularly, the modules may help understand the mechanisms of sJIA, and the hub genes may become biomarkers and therapeutic targets of sJIA in the future.

## 1. Introduction

Systemic juvenile idiopathic arthritis (sJIA) is a serious immune inflammatory pediatric disorder that is markedly different from other JIA subtypes in many aspects. Symptoms for patients with sJIA range from fever, rash, serositis, lymphadenectasis, liver and/or spleen enlargement [1], and the potentially life-threatening macrophage activation syndrome (MAS). It is estimated that about 10% of patients with sJIA will develop overt MAS, and more than 50% of the patients may have subclinical MAS [2, 3]. sJIA shares many characteristics with classic autoinflammatory diseases, most notably the response to IL-1 inhibition [4]. It is associated with other inflammatory cytokines, such as IL-6 and IL-18 and the S100 alarm protein [5]. Besides, NK cell dysfunction has been suggested as a common pathway in patients with sJIA, MAS, and HLH (hemophagocytic lymphohistiocytosis, closely resembling MAS [6, 7]). However, the molecular basis of sJIA immune dysfunction and the etiology of sJIA and MAS remain poorly understood [8], as many genetic and genomic investigations on sJIA are limited due to the small sample size.

Weighted gene coexpression network analysis (WGCNA) is a network-based approach that focuses on sets of genes instead of individual genes from gene expression data. By transforming the data of the gene expression into coexpression modules, WGCNA provides insights into key genes and signaling networks that could play critical roles in the progression of diseases [9–11]. This approach has been extensively used in biological research, such as that involving cancer [12], chronic obstructive pulmonary disease (COPD) [13], and neuropsychiatric disorders [14]. WGCNA is a powerful tool for screening candidate biomarkers or therapeutic targets. In this study, based on the integrated microarray datasets, we used the WGCNA method to identify sJIA-related and non-sJIA coexpression modules and analyzed the hub genes in the modules. The biological functions and pathways of the two modules were also identified and analyzed. To the best of our knowledge, this is the first study to apply the WGCNA method to multiple datasets to understand the molecular mechanism of sJIA.

## 2. Materials and Methods

### 2.1. Data Collection

**Figure 1:**
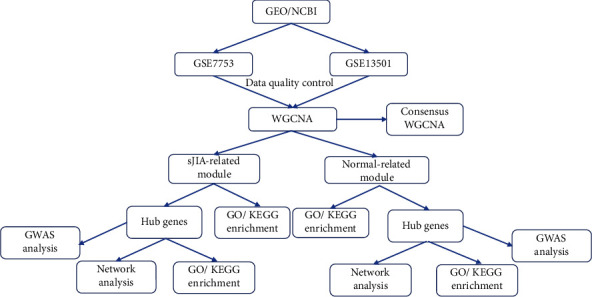
Flow chart of the whole procedures in this study.

Gene profiles were downloaded from the Gene Expression Omnibus (GEO) database (https://www.ncbi.nlm.nih.gov/geo/). The inclusion criteria keywords were as follows: (1) systemic juvenile idiopathic arthritis, (2) Homo sapiens, and (3) peripheral blood tissue. Datasets with drug stimulation or transfection were excluded. Finally, we selected GSE7753 [15] and GSE13501 [16] as these were the only two datasets meeting the criteria; this step was performed using the same platforms (Affymetrix Human Genome U133 Plus 2.0 Array GPL570). GSE7753 contained 17 sJIA samples and 30 normal samples; GSE13501 included 21 sJIA samples and 59 normal samples. The raw data of GSE7753 [15] and GSE13501 [16] were downloaded from the GEO database. Altogether, 127 samples (38 sJIA and 89 healthy controls) were used in the analysis. The overall search process is illustrated in Figure 1.

The affy package (*R* environment, version 3.6.1) was used to normalize (RMA normalization) and preprocess the raw data [17]. The parameters were set as RMA (for background correction) and impute (for supplemental missing values). The expression profiles were log2 transformed, and batch normalization was performed using “sva” and “combat” functions in SVA *R* package [18], in order to avoid a possible bias of the two separate microarray datasets. Probes with more than one gene were eliminated, and the maximum value was selected from these probes after probe annotation. A series matrix file was preprocessed to identify differentially expressed genes based on variance analysis, and the top 25% [19] (5,414 genes) was obtained for subsequent analysis.

### 2.2. Construction of the Weighted Coexpression Network

The “WGCNA” package in *R* software was used for the network construction [11]. The expression values of the 5,414 genes were imported into WGCNA to construct coexpression modules using automatic network construction. The pickSoftThreshold function was used to calculate the scale-free topology fit index for 1 to 20 powers, and a soft threshold power of six was chosen as the most appropriate one for network construction. Then, automatic block-wise module detection was performed using the function blockwiseModules. The function first preclustered the nodes into large clusters. Then, hierarchical clustering is applied to each block, and the modules are defined as branches of the resulting dendrogram. An automatic module merging step is performed to merge modules whose eigengenes were highly correlated (maxBlockSize = 6000, TOMType = ^“^unsigned,^”^minModuleSize = 40, mergeCutHeight = 0.25). Thus, genes with similar expression profiles were separated into the same module.

### 2.3. Construction of the Consensus-Weighted Coexpression Network

The consensus WGCNA method was applied to verify the reliability and stability of the previous results and the module, and GSE7753 and GSE13501 were named as sJIA1 and sJIA2 datasets, respectively, for subsequent analysis. It was also important to choose the soft-thresholding power *β* to construct a consensus-weighted gene network. An approximate scale-free topology was attained around the soft-thresholding power of 6 for both sets. The parameters were set as follows: the soft-thresholding power 6, minimum module size 40, and cut height for merging of modules 0.25, implying that modules whose eigengenes are correlated above 1 − 0.25 = 0.75 will be merged, and 17 distinct gene coexpression modules were constructed and shown in different colors. Consensus modules were also related to external microarray sample information, sJIA patients, and healthy individuals. In addition, the correspondences were compared among individual dataset modules, merged dataset modules, and consensus modules.

### 2.4. Identification of Coexpression Modules Related to sJIA and Non-sJIA Samples

The associations between the module and trait were estimated with the correlation between the module eigengene and the clinical traits, namely, sJIA and non-sJIA. Here, gene significance (GS) is defined as the absolute value of the correlation between the gene and the trait, and module membership (MM) represents the correlation of the genes with each module eigengene and clinical feature. Furthermore, module importance (MS) is defined as the correlation between the module eigengene and gene expression profile. Among all coexpression modules, the module with the absolute MS ranking first was regarded as a module related to clinical traits (sJIA module and non-sJIA module). The gene modules with the highest correlation to non-sJIA and sJIA were selected for subsequent studies.

### 2.5. Function Enrichment Analysis

Functional enrichment analysis was performed on the genes in the sJIA and non-sJIA modules. Information on the module genes was submitted to Database for Annotation, Visualization, and Integrated Discovery (DAVID) online tool (https://david.ncifcrf.gov/) [20] to perform functional annotation based on Gene Ontology (GO) [21] and Kyoto Encyclopedia of Genes and Genomes (KEGG) pathway analyses. The packages anRichment and anRichmentMethods were used to perform GO enrichment analysis in all modules (https://horvath.genetics.ucla.edu/html/CoexpressionNetwork/GeneAnnotation/Tutorials/). The packages were developed by the inventor of WGCNA, which helped evaluate the enrichment of the gene modules in the collection of GO terms (threshold = 1*e* − 4, thresholdType = ^“^Bonferroni^”^), and selected the top GO results in each module to draw a bar graph (Supplementary 1 Figure S4).

### 2.6. Identification of Hub Genes

Hub genes are considered functionally significant because of their high connectivity with other genes within a module. In this study, the 30 top ranked genes with the highest levels of intramodular connectivity in the two modules were selected as candidates for further analysis using DAVID and visualized using Cytoscape. Subsequently, the genome-wide association study (GWAS) catalog (https://www.ebi.ac.uk/gwas/studies/GCST004025) was used to obtain the disease-susceptibility genes identified by a previously published GWAS [22]. The protein-protein interaction (PPI) network of the module hub genes and GWA genes was analyzed using the STRING (https://string-db.org/) database (confidence score ≥0.4) and visualized using Cytoscape. Comparative analyses of the functional enrichment among the module hub genes and GWA genes were performed using the online bioinformatic database Metascape (http://metascape.org/gp/index.html#/main/step1) [23].

## 3. Results

### 3.1. Construction of the Weighted Coexpression Network

**Figure 2:**
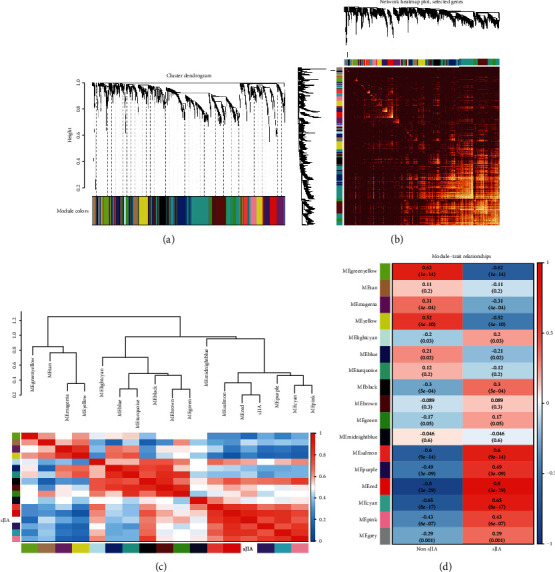
Division and validation of coexpression modules. (a) Dendrogram of all genes divided into 17 modules, with dissimilarity based on topological overlap, together with assigned module colors. The number of genes in each module was listed in Supplementary 1 Table S2. (b) The heat map depicts the topological overlap matrix (TOM) among all genes in the analysis. The depth of the red color indicates the correlation between all pair-wise genes. (c) The upper part shows hierarchical clustering of the whole modules. Another is a heat map plot of the adjacencies in the hub gene network. (d) Heat map of the correlation between module eigengenes and the clinical modules.

After data preprocessing, a total of 5,414 genes were selected for WGCNA. First, an appropriate soft-thresholding power of 6 was selected (Supplementary 1 Figure S1–S2 and Supplementary 1 Table S1) and used to construct the coexpression module. Seventeen distinct gene coexpression modules were constructed shown in different colors in Figure 2(a). The number of genes in the 17 modules is shown in Supplementary 1 Table S2.  Figure 2(b) shows the topological overlap matrix (TOM) of the 5,414 genes, indicating that each module and gene expression in each module was relatively independent. Furthermore, we plotted the clustering dendrogram; according to the module correlation and the heat map according to adjacencies (Figure 2(c)), indicating that these modules were largely divided into two clusters.

### 3.2. Construction of the Consensus Weighted Coexpression Network

As the overall connectivity index generally drops sharply with an increase in the soft-thresholding power, it is advantageous to select the lowest power that meets the approximate scale-free topology standard. As shown in Supplementary 1 Figure S2–S3, an approximate scale-free topology was obtained around the soft-thresholding power of 6 for both sets.

**Figure 3:**
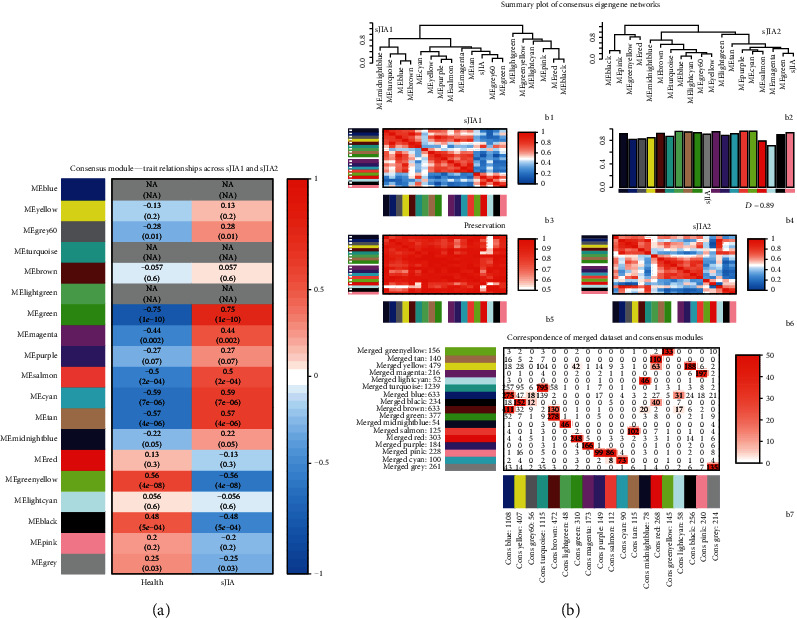
Correspondence among individual dataset modules, merged dataset modules, and consensus modules. (a) Heat map of the correlation between module eigengenes and the clinical modules. Missing (NA) entries indicate that the correlations in the sJIA1 and sJIA2 datasets have opposite signs, and no consensus can be formed. (b, B1–B6): Correspondence of the sJIA1 dataset modules and sJIA2 modules. The Preservation heat map shows the preservation network, defined as one minus the absolute difference of the eigengene networks in the two datasets. The bar plot shows the mean preservation of adjacency for each of the eigengenes to all other eigengenes. (b, B7): Correspondence of the merged dataset modules and consensus modules. Each row of the table corresponds to one sJIA2 set-specific module, and each column corresponds to one consensus module. Numbers in the table indicate gene counts in the intersection of the corresponding modules.

Seventeen different gene coexpression modules were constructed, shown in different colors in Figure 2(a) and related to external microarray sample information, patients with sJIA, and healthy individuals. In each of the two sets, consensus module eigengenes were related to the traits. To summarize the two sets into one measure of module-trait relationships, we selected the correlation that had the lowest absolute value in the two sets if the two correlations had the same sign and zero relationship if the two correlations had opposite signs (Figure 3(a)). We checked these genes in the modules related to clinical features and observed results consistent with our previous results.

We then compared the correspondence among individual dataset modules, merged dataset modules, and consensus modules.  Figure 3B1–B6 show that the two datasets are indeed very similar. The preservation heat map and bar plots indicate that most relationships were very highly preserved, and the overall preservation of the two eigengene networks was 0.89.  Figure 3B7 shows that the number of genes overlapping between the merged dataset modules (our previous method) and consensus modules was extremely high, and the hub genes obtained in the previous WGCNA modules were all overlapping genes.

### 3.3. Identification of Coexpression Modules Related to Non-sJIA or sJIA Samples

**Figure 4:**
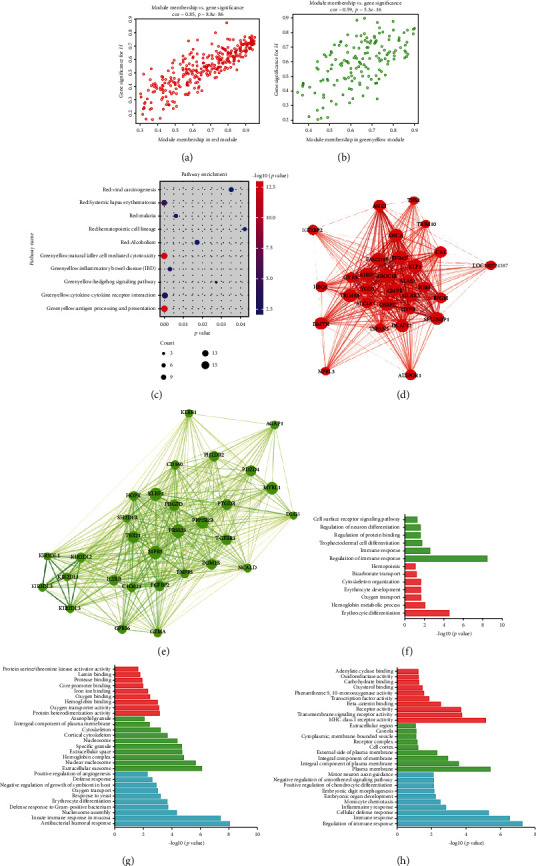
Functional enrichment analyses of two trait-related modules. (a) Scatter plot of module eigengenes in the red module. (b) Scatter plot of module eigengenes in the greenyellow module. (c) KEGG enrichment analyses of two trait-related modules. (d) The top 30 hub genes in the red module. (e) The top 30 hub genes in the green-yellow module. Nodes represent genes, and node size is correlated with connectivity of the gene. (f) GO enrichment analyses of the top 30 hub genes in the two trait-related modules. (g) GO enrichment analyses of the red module. (h) GO enrichment analyses of the green-yellow module.

The module-trait correlation coefficients showed that the red module and green-yellow module were highly correlated with disease status (Figure 2(d)). The red module was positively correlated with the sJIA-related module (*r* = 0.8, *p* = 3*e*^−29^), whereas the green-yellow module was negatively correlated to the sJIA (*r* = 0.62, *p* = 1*e*^−14^). The scatterplots in Figures 4(a) and 4(b) show that the gene significance (GS) and module membership (MM) values were highly correlated in the red module (cor = 0.85, *p* = 8.8*e*^−86^) and the green-yellow module (cor = −0.59, *p* = 5.3*e*^−16^), suggesting that the genes in these two modules were probably related to the disease status. Thus, the red module was defined as the sJIA-related module, and the green-yellow module was defined as the non-sJIA module, which was suitable for further analyses and mining of the hub gene.

### 3.4. Function Enrichment Analysis

Functional enrichment analysis conducted using DAVID was performed on the genes in the two constructed modules. There was a significant difference in the biological processes of genes in the sJIA and non-sJIA modules. The detailed information is displayed in Figures 4(g) and 4(h) and Supplementary 1 Table S3-S4.

For the red module, GO biological process (BP) annotation showed that the gene products were mainly enriched in activation of immune response, infection, nucleosome, and erythrocyte. Regarding GO molecular function (MF) annotation, protein heterodimerization and oxygen transporter were the most enriched terms. Enriched GO-CC terms were mainly involved in extracellular exosome, nucleosome, hemoglobin complex, and extracellular space. The results of KEGG enrichment analysis showed that the module was similar to that of systemic lupus erythematosus (SLE) (gene count = 11, *p* = 3.1*e*^−5^). For the green-yellow module, GO-BP annotation was mainly enriched in the immune response and inflammation. Receptor activity was the top enriched GO-MF terms, with the plasma membrane enriched in GO-CC terms. Similarly, the KEGG terms were mainly related to “antigen processing and presentation” and “natural killer cell-mediated cytotoxicity” (Figure 4(c)).

**Figure 5:**
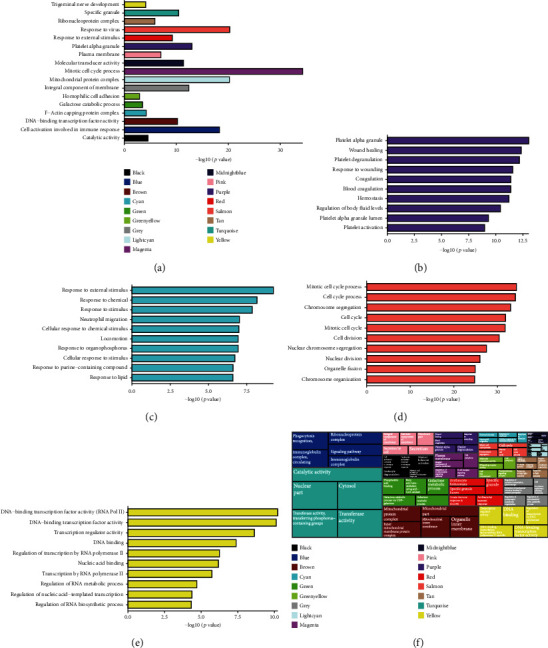
GO enrichment analysis of each module in WGCNA. (a) Bar plot of the top GO terms of each module. The bars are marked with the corresponding module color, and the left side is the specific GO terms. (b–e) Bar plot of GO enrichment analysis in other relatively important modules: yellow, salmon, purple, and cyan. (f) Treemap of the top five GO terms of each module.

We also used the packages anRichment and anRichmentMethods to perform GO enrichment analysis in the whole module and select the top GO term in each module to draw a bar graph (Figures 5(a) and 5(f)). We further analyzed the functional enrichment of genes in several other relatively important modules: yellow, salmon, purple, and cyan. As shown in Figures 5(b) – 5(e), the cyan module was mainly related to the response to external stimuli; the purple module was mostly related to the function of platelet alpha granules, involving pathways such as wound healing, coagulation, and hemostasis; the salmon module may play an important role in the cell cycle process, and the yellow module was associated with transcriptional regulation.

### 3.5. Identification of Hub Genes

The 30 top ranked hub genes in the two modules are shown in Cytoscape (Figures 4(d) and 4(e) and Supplementary 1 Table S5-S6). As shown in Figure 4(f), the hub genes from the red module were largely related to erythrocyte differentiation (*ALAS2*, *AHSP*, *KLF1*, *TRIM10*, and *TRIM58*), and the hub genes from the green-yellow module were largely involved to immune responses, exemplified by genes such as *KLRB1*, *KLRF1*, *CD160*, *KIR2DL1*, *KIR2DL2*, *KIR2DL3*, *KIR3DL1*, *SH2D1B*, *GZMA*, and *TGFBR3* (Supplementary 1 Table S7).

**Figure 6:**
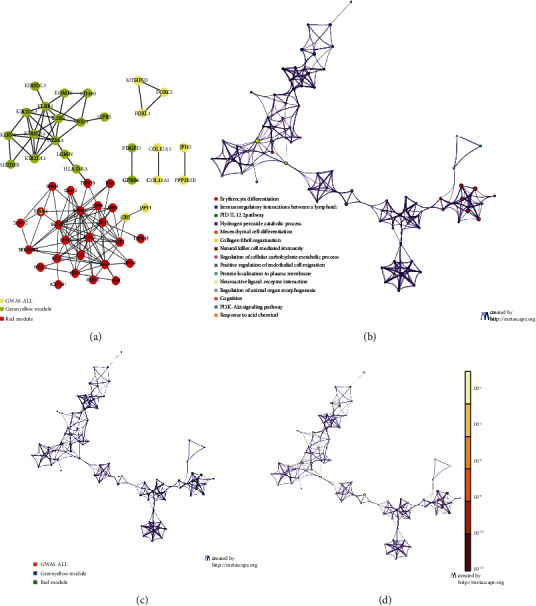
Interactions between module hub genes and genome-wide-associated genes. (a) Protein-protein interaction network of the module hub genes and genome-wide-associated genes. Common genes between GWAS and the “red” module are denoted in yellow nodes with red contour. (b) Enriched ontology clusters colored by cluster ID. Terms with a similarity score > 0.3 are linked by an edge (the thickness of the edge represents the similarity score). (c) Enriched ontology clusters pied by gene counts across studies. Each pie sector is proportional to the number of hits originated from a gene list. Color code for pie sector represents a gene list. (d) Enriched ontology clusters colored by *p* value. The dark the color, the more statistically significant the node is.

Only one GWAS study was previously conducted on sJIA [22], and we obtained the disease-susceptibility genes from this manuscript and the GWAS catalog. To further compare the hub genes with the GWA genes, we performed PPI network analysis and functional enrichment. As shown in Figure 6(a), the sJIA-susceptible genes (*HLA-DRA*, *TRIM58*, *LDB2*, and *TAPT1*) may be related to the hub genes from the red module, with *TRIM58* being a hub gene of the red module, and *LGMN* and *JPH3* may be related to the hub genes from the green-yellow module. Figures 6(b) – 6(d) show the comparative analyses of the functional enrichment, such as tissue morphogenesis related to GWA genes and the hub genes of the red module, endothelial cell migration associated with GWA genes, and the hub genes of the green-yellow module (Supplementary 1 Figure S5A-S5C).

## 4. Discussion

Bioinformatic analysis of the public gene expression data could provide further knowledge on the pathogenesis of sJIA. This is the first study to integrate multiple datasets and construct WGCNA to identify hub genes that may play an important role in sJIA. Among the 17 coexpression modules, the red module was positively related to sJIA, and the green-yellow module was negatively related to sJIA. Moreover, we identified several hub genes related to the pathogenesis of sJIA. As the genes in the same module were considered to have similar functions, the analysis of biologically relevant modules and hub genes may provide new insights into the molecular mechanism of sJIA development.

The red module was critical in biological processes and pathways such as antibacterial humoral response, innate immune response in mucosa, nucleosome assembly, defense response to gram-positive bacteria, and erythrocyte differentiation. However, the functional enrichment of the top 30 hub genes in the red module was largely related to erythrocyte differentiation (*ALAS2*, *AHSP*, *TRIM10*, *TRIM58*, and *KLF1*). In accordance with the present results, previous studies [24] have demonstrated that there is a strong relationship between the erythroid differentiation signature (EDS) and sJIA associated with the expansion of CD34+ cells. The presence of EDS was also observed in familial hemophagocytic lymphohistiocytosis (fHLH), infection, and pulmonary arterial hypertension (PAH), suggesting that the increased recruitment of red blood cells might be a part of the systemic response to severe chronic local hypoxia [24, 25].

*ALAS2* (erythroid-specific 5-aminolevulinate synthase) is the first and rate-limiting enzyme in the erythroid heme biosynthetic pathway [26]. Mutations in *ALAS2* may be related to porphyria and X-linked sideroblastic anemia [27]. *AHSP* (alpha hemoglobin–stabilizing protein) is also necessary for the proper assembly of nascent alpha-globin into hemoglobin-A [28]. The altered expression or function of *AHSP* might be related to the severity of thalassemia [29]. A recent study by Lechauve et al. also predicted that *AHSP* plays an important role in the physiological process of regulating vascular NO concentration [30]. *KLF1* (erythroid Kruppel-like factor 1) is important in the function of erythroid cells, such as red cell membrane stability and heme biosynthesis [31]. *AHSP* is also a known as the KLF1 target gene, the expression of which is significantly upregulated upon *KLF1* activation [31]. The protein encoded by *LDB2* (LIM domain binding 2) belongs to the LIM domain binding family that also play critical roles in cell fate determination, differentiation, and tissue development [32]. Further studies are required to confirm and validate the function of these EDS genes (*ALAS2*, *AHSP*, *KLF1*, and *LDB2*) in the occurrence and development of diseases.

*TRIM* family proteins play an essential and unique role in several diseases, such as immunological diseases, cancers, and developmental disorders, and may function as dual regulators of the immune response and carcinogenesis [33]. *TRIM10* has been reported to participate in terminal red blood cell differentiation and survival [34]. However, recent research has shown that *TRIM10* is involved in Parkinson's disease (PD) and other autoimmune diseases [35]. Silencing of *TRIM10* reduced apoptosis and reactive oxygen species levels in a cellular model of PD, which suggests a potential role of *TRIM10* in PD and other autoimmune diseases. An earlier study also revealed the role of *TRIM58* in the regulation of human erythrocyte traits [36]. Recent studies have reported that *TRIM58* regulates epithelial–mesenchymal transition (EMT) via the Wnt/*β*-catenin pathway [37] and may function as a tumor suppressor in some cancers, such as colorectal cancer [37] and gastric cancer [38]. Another study [39] showed that *TRIM58* might protect against the transduction of intestinal mucosal inflammation by inhibiting abnormal TLR2 signaling and serve as a potential therapeutic target in autoimmune diseases, such as ulcerative colitis. Furthermore, *TRIM58* was identified as an sJIA susceptibility gene in a previous GWAS on sJIA [22].

For the green-yellow module, function enrichment analysis mainly identified the immune response and inflammation pathways, and the results of the hub genes were similar. The hub genes from the green-yellow module were largely related to immune responses, exemplified by genes such as *KLRB1*, *KLRF1*, *CD160*, *KIR2DL1*, *KIR2DL2*, *KIR2DL3*, *KIR3DL1*, *SH2D1B*, *GZMA*, and *TGFBR3*, which was in line with previous studies showing that NK cell dysfunction may be a common pathway in sJIA, MAS, and HLH [6, 7]. Moreover, inflammatory driver factors may be involved in the cytotoxic effects of NK cells in MAS and sJIA [7].

*KLRB1* (killer cell lectin-like receptor subfamily B member 1), usually referred to as CD161, is a type II transmembrane C-type lectin glycoprotein that appears to play an inhibitory role in IFN-*γ* secretion [40] and on human NK cells [41], while its function on T cells remains elusive, with reports suggesting both coactivating [40] and inhibitory [42] effects. *KLRB1* has been previously shown to be downregulated in rheumatoid arthritis [43] and SLE [44–46].

*KLRF1* (killer cell lectin-like receptor F1) is an activated homodimeric C-type lectin-like receptor (CTLR) expressed on most NK cells, marking a critical step in human NK cell development [47] and stimulates cytotoxicity and cytokine release by the NK cells [48]. CD160 (a 27-kDa glycoprotein) tightly binds to peripheral blood NK cells and CD8^+^ T lymphocytes and has a cytolytic effect [49]. The killer cell immunoglobulin-like receptor (KIR) family of inhibitory receptors, which includes *KIR2DL1*, *KIR2DL2*, *KIR2DL3*, and *KIR3DL1*, plays the most important role in NK cell activation [50]. A previous study [51] showed that sJIA, compared with poly and pauciarticular JIA, was related to the decreased NK cell function, with more IFN-*γ*, less TNF-*α* secretion by NK cells, and lower *KIR2DS4* levels. Further and larger studies on the KIR gene family are necessary. Moreover, another hub gene, *SH2D1B* (SH2 domain containing 1 B), also plays a key role in the regulation of effector functions of NK cells by controlling signal transduction through CD244/2B4 [52].

The protein encoded by *GZMA* (granzyme A), which belongs to the granzyme family [53], lyses target cells through cytotoxic T lymphocytes and NK cells. Multiple studies have reported that the cytotoxicity of NK cells in the peripheral blood mononuclear cells (PBMCs) of patients with sJIA is reduced. A previous study [51] showed that patients with sJIA have lower granzyme B expression levels (*p* < 0.05), whereas patients with poly- and pauciarticular JIA have higher perforin and granzyme B expression levels (*p* < 0.05). Another study [54] also found decreased expression of granzyme K in CD56^bright^ NK cells at the protein and transcriptional levels. However, the intrinsic cytotoxic defect in sJIA remains undetermined [7], and the action of *GZMA* is not clearly understood.

Our study has several merits, the most obvious being that it is the first study to integrate multiple datasets and apply the WGCNA method to understand the molecular mechanisms of sJIA. Due to the rarity of the disease, we were unable to obtain a larger number of samples; nevertheless, we tried our best to obtain all available data. We not only merged multiple datasets but also used a consensus WGCNA to prove that our conclusions are conservative and reliable in multiple datasets. Previous research on the disease mainly focused on blood leukocytes, such as the immensely innovative and pioneering study by Cepika et al. [55], which integrated the blood leukocyte responses to innate stimuli from multiple omics, and determined the gene set related to specific cytokine environment and activated leukocyte subsets in sJIA. However, our study found a relatively novel mechanism of sJIA in red blood cell differentiation [24] and NK cell disorder [6, 7]. Furthermore, by linking the susceptibility genes with the module-associated hub genes, we improved our understanding on the biological processes in sJIA and identified TRIM58 both as an sJIA susceptibility gene and as a hub gene of the red module. There is still a large gap in the knowledge regarding the occurrence and development of sJIA. Therefore, we consider that our study may help to investigate the progress of sJIA, and that hub genes may become biomarkers and therapeutic targets of sJIA in the future.

## 5. Conclusion

In conclusion, we identified sJIA-associated key genes, such as *ALAS2*, *AHSP*, *TRIM10*, *TRIM58*, and*KLF1*, which are largely related to erythrocyte differentiation. These genes may be related to anemia or MAS in sJIA. *KLRB1*, *KLRF1*, *CD160*, and *KIRs* might be related to NK cell dysfunction, which has been studied extensively but remains poorly understood in the context of sJIA pathogenesis. Our study holds implications in understanding the progression and development of sJIA, and the identified hub genes may serve as biomarkers and therapeutic targets of sJIA in the future.

## Data Availability

Publicly available datasets were analyzed in this study. This data can be downloaded from here: https://www.ncbi.nlm.nih.gov/geo/query/acc.cgi?acc=GSE7753 and https://www.ncbi.nlm.nih.gov/geo/query/acc.cgi?acc=GSE13501.

## Abbreviations

sJIA: Systemic juvenile idiopathic arthritis
WGCNA: Weighted gene coexpression network analysis
MAS: Macrophage activation syndrome
HLH: Hemophagocytic lymphohistiocytosis
COPD: Chronic obstructive pulmonary disease
GS: Gene significance
MM: Module membership
MS: Module importance
DAVID: Database for Annotation, Visualization, and Integrated Discovery
GO: Gene Ontology
KEGG: Kyoto Encyclopedia of Genes and Genomes
BP: Biological process
MF: Molecular function
PPI: Protein-protein interaction
fHLH: Familial hemophagocytic lymphohistiocytosis
PAH: Pulmonary arterial hypertension
*ALAS2*: Erythroid-specific 5-aminolevulinate synthase
*AHSP*: Alpha hemoglobin–stabilizing protein
*KLF1*: Kruppel-like factor 1
*KLRB1*: Killer cell lectin-like receptor subfamily B member 1
*KLRF1*: Killer cell lectin-like receptor F1
*KIRs*: Killer cell immunoglobulin-like receptors.

## Supplementary Materials

Supplementary Materials Supplementary 1 Figure S1: sample clustering to detect outliers. Figure S2: analysis of network topology for various soft-thresholding powers. Figure S3: construction of the consensus WGCNA. Figure S4: bar plot of mean gene significance across modules. Figure S5A-S5C: interactions between module hub genes and genome-wide-associated genes. Table S1: analysis of network topology for various soft-thresholding powers. Table S2: the number of genes in each module. Table S3: the GO and KEGG pathway enrichment analysis of the red module. Table S4: the GO and KEGG pathway enrichment analysis of the green-yellow module. Table S5: the hub genes in the red module. Table S6: the hub genes in the green-yellow module. Table S7: the GO enrichment analysis of hub genes in red and green-yellow module. Supplementary 2 WGCNA codes.
