# Supplementary material for: Application of Weighted Gene Coexpression Network Analysis to Identify Key Modules and Hub Genes in Systemic Juvenile Idiopathic Arthritis
Source: Biomed Res Int. 2021 Aug 13;2021:9957569. doi: 10.1155/2021/9957569 (PMC8382540; doi:10.1155/2021/9957569)
Supplement: Supplementary Materials — Supplementary 1 Figure S1: sample clustering to detect outliers. Figure S2: analysis of network topology for various soft-thresholding powers. Figure S3: construction of the consensus WGCNA. Figure S4: bar plot of mean gene significance across modules. Figure S5A-S5C: interactions between module hub genes and genome-wide-associated genes. Table S1: analysis of network topology for various soft-thresholding powers. Table S2: the number of genes in each module. Table S3: the GO and KEGG pathway enrichment analysis of the red module. Table S4: the GO and KEGG pathway enrichment analysis of the green-yellow module. Table S5: the hub genes in the red module. Table S6: the hub genes in the green-yellow module. Table S7: the GO enrichment analysis of hub genes in red and green-yellow module. Supplementary 2 WGCNA codes. [file 9957569.f1.zip › 9957569.f1/Supplementary1_simple figure and table_ updated.docx]

Supplementary1

**Figure S1 |** Sample clustering to detect outliers.

**Figure S2 |** Analysis of network topology for various soft-thresholding powers.

**Figure S3 |** Construction of the Consensus WGCNA.

**Figure S4 |** Barplot of mean gene significance across modules.

**Figure S5A- S5C |** Interactions between module hub genes and genome-wide-associated genes.

**TABLE S1 |** Analysis of network topology for various soft-thresholding powers.

**TABLE S2 |** the number of genes in each module.

**TABLE S3 |** The GO and KEGG pathway enrichment analysis of red module.

**TABLE S4 |** The GO and KEGG pathway enrichment analysis of greenyellow module.

**TABLE S5 |** The hub genes in the red module.

**TABLE S6 |** The hub genes in the greenyellow module.

**TABLE S7 |** The GO enrichment analysis of hub genes in red and greenyellow module.


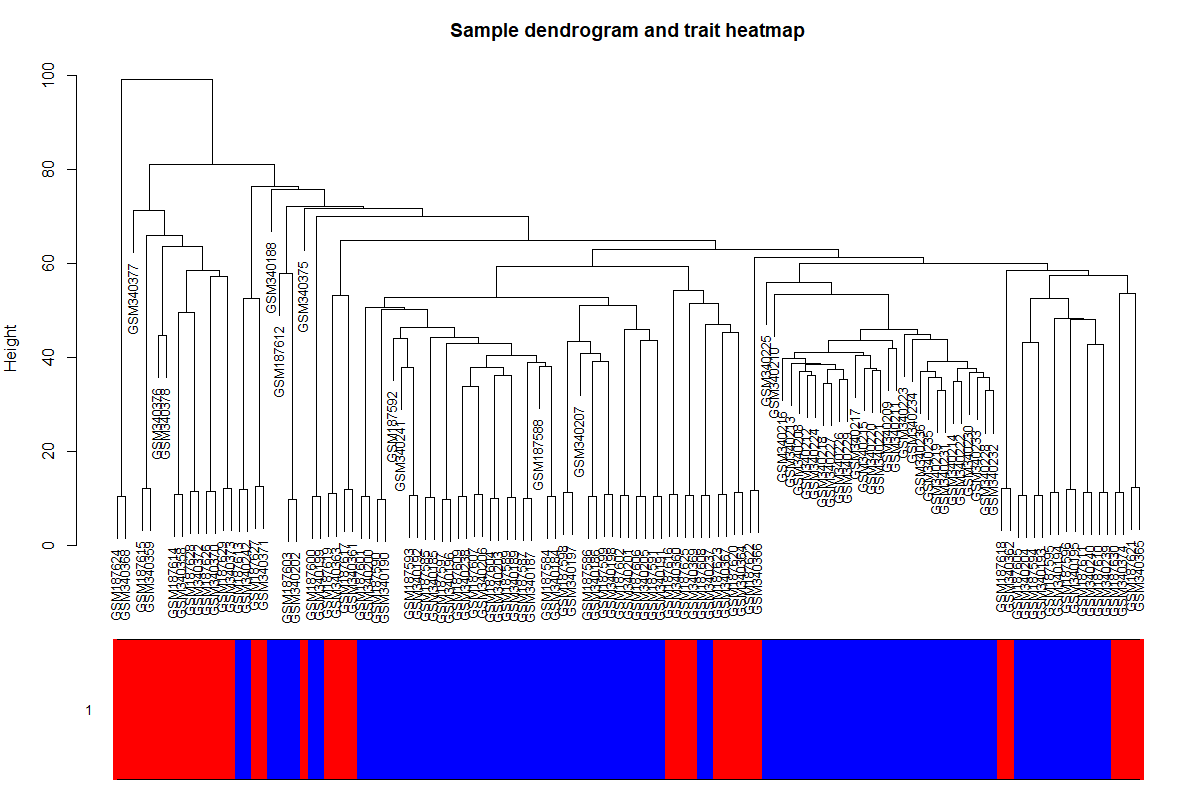


Supplementary Figure S1: Sample clustering to detect outliers. All the samples were in the clusters，all samples have passed the cuts.


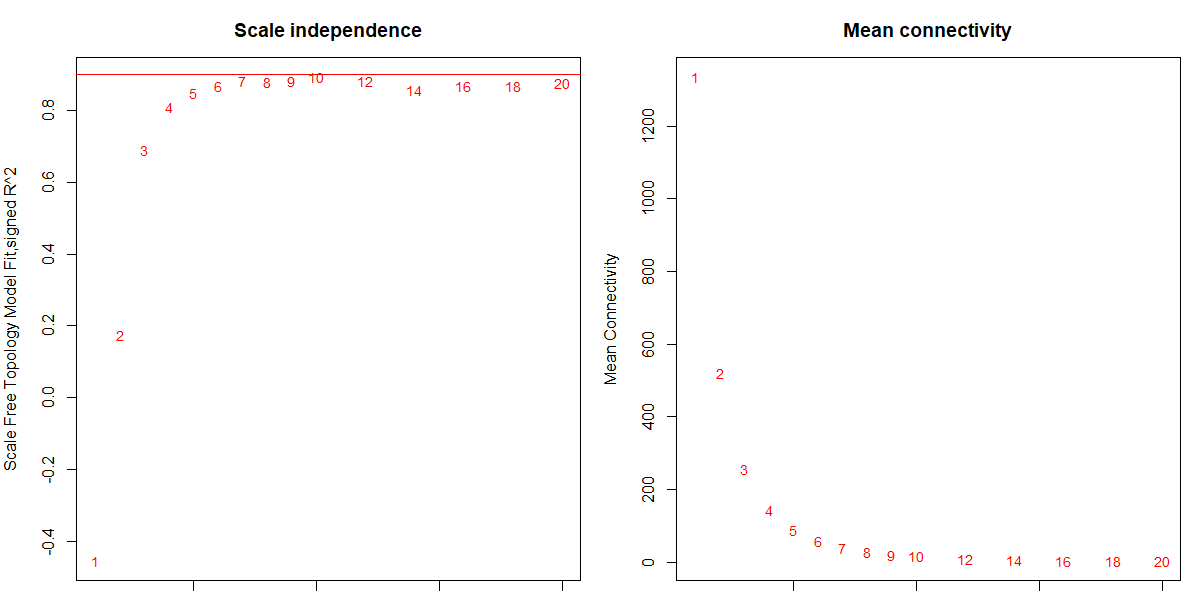


Supplementary Figure S2: Analysis of network topology for various soft-thresholding powers. The left panel shows the scale-free fit index (y-axis) as a function of the soft-thresholding power (x-axis). The right panel displays the mean connectivity (degree, y-axis) as a function of the soft-thresholding power (x-axis).


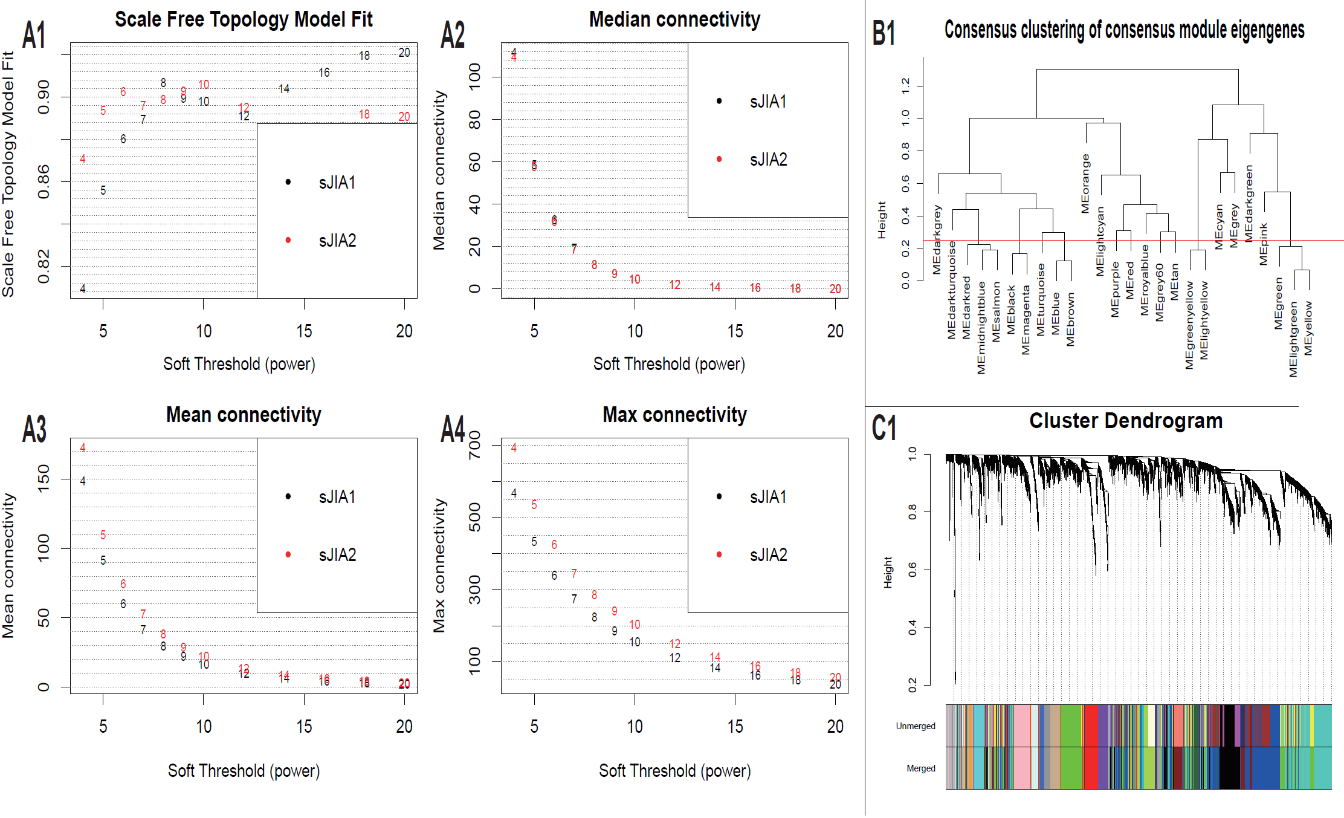


Supplementary Figure S3: Construction of the Consensus WGCNA: selection of the soft thresholding power and Construction of Consensus Modules. (A1-A4): Summary network indices as functions of the soft thresholding power. Numbers in the plots indicate the corresponding soft thresholding powers. (B): Clustering of consensus module eigengenes. (C). Dendrogram of all genes divided into 19 modules, with dissimilarity based on topological overlap, together with assigned module colors.


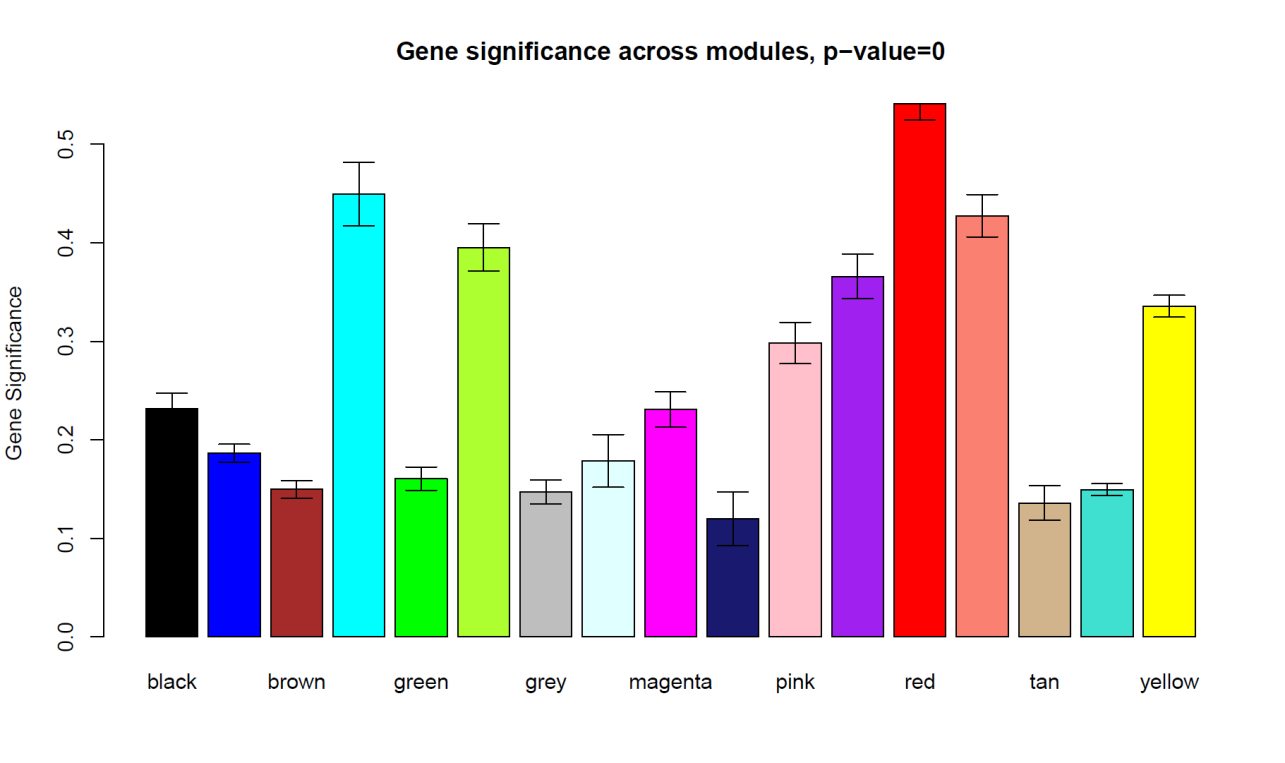


Supplementary Figure S4: Barplot of mean gene significance across modules. The higher the mean gene significance in a module, the more significantly related the module is to the clinical trait of interest.


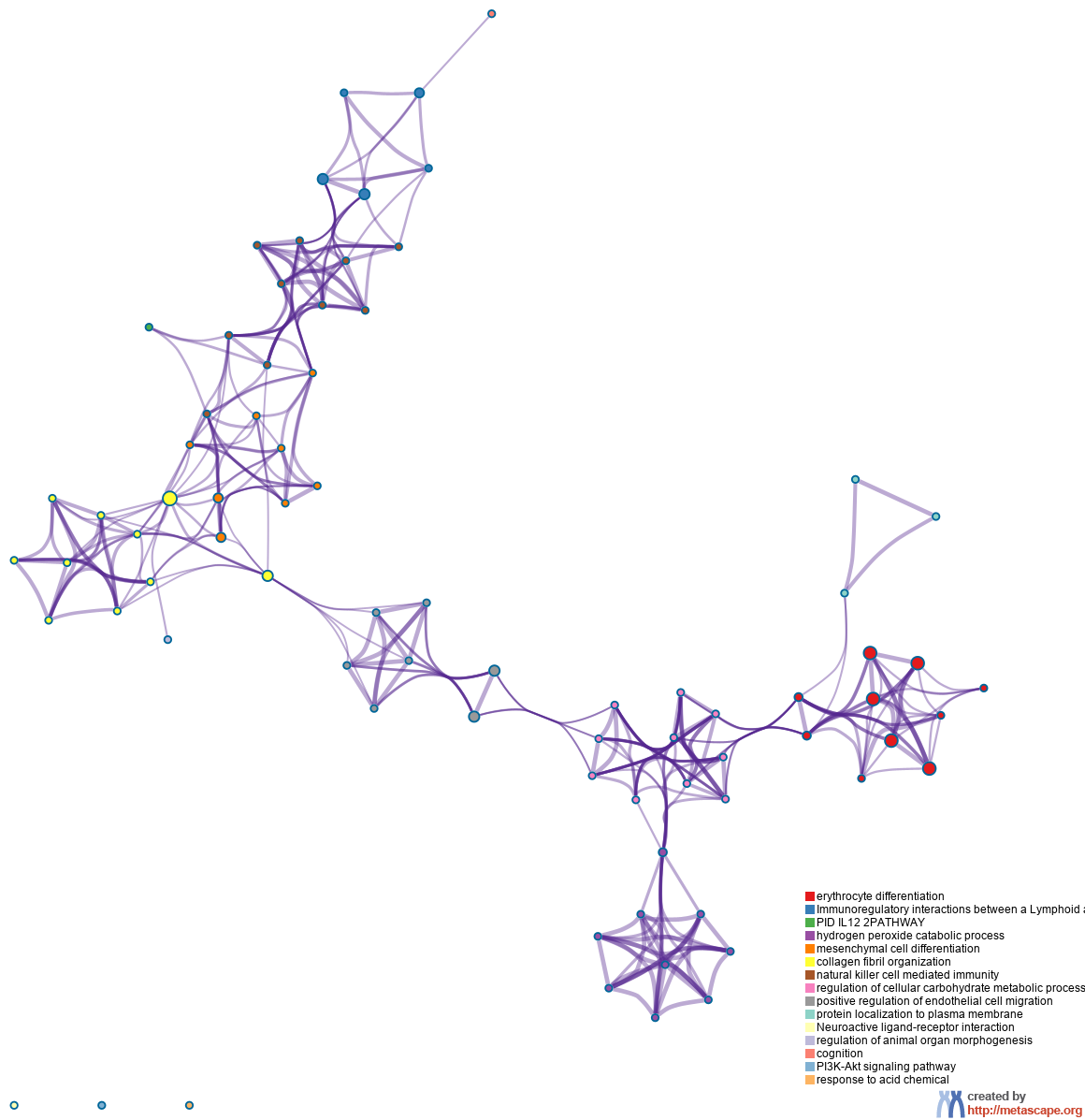


Figure S5A | Interactions between module hub genes and genome-wide-associated genes.

A. Enriched Ontology Clusters Colored by Cluster ID. Terms with a similarity score > 0.3 are linked by an edge (the thickness of the edge represents the similarity score).


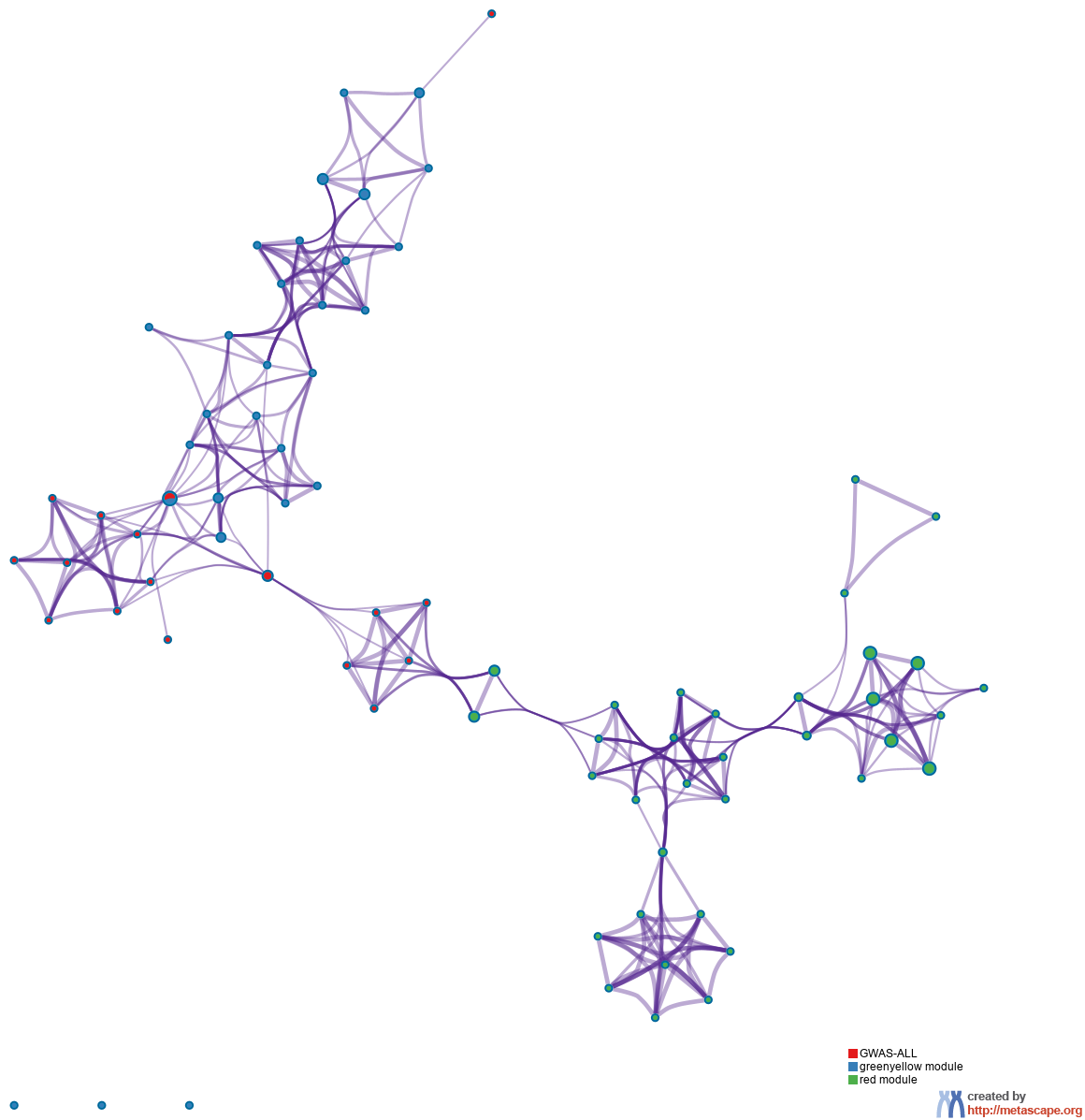


Figure S5B | Interactions between module hub genes and genome-wide-associated genes.

B. Enriched Ontology Clusters Pied by Gene Counts Across Studies. Each pie sector is proportional to the number of hits originated from a gene list. Color code for pie sector represents a gene list.


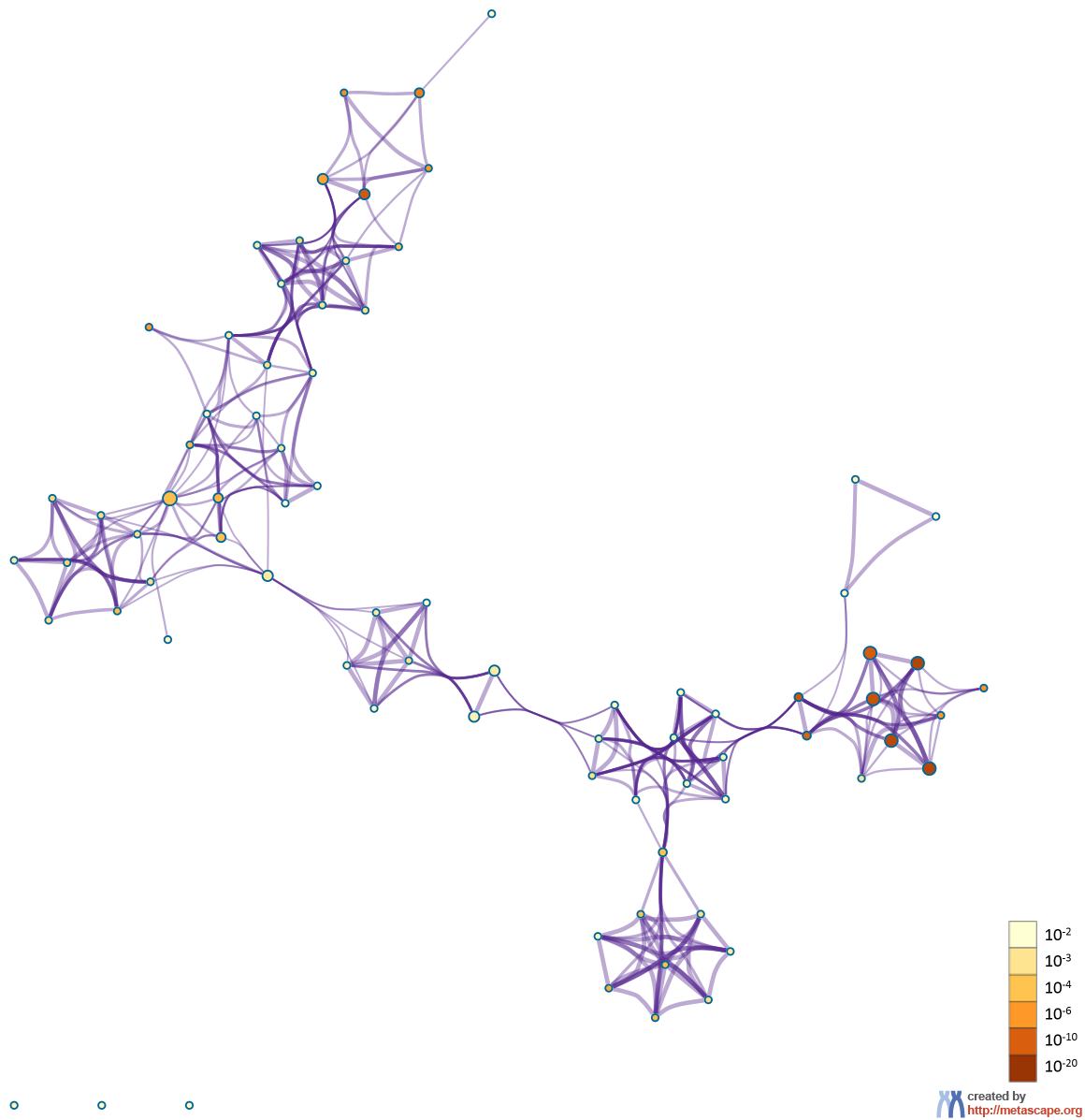


Figure S5C | Interactions between module hub genes and genome-wide-associated genes.

C. Enriched Ontology Clusters Colored by p-Value. The dark the color, the more statistically significant the node is.

TABLE S1 Analysis of network topology for various soft-thresholding powers.

| Power | SFT.R.sq | slope | truncated.R.sq | mean.k. | median.k. | max.k. |
| --- | --- | --- | --- | --- | --- | --- |
| 1 | 0.454984 | 1.283887 | 0.956456 | 1335.557 | 1341.804 | 2117.693 |
| 2 | 0.17479 | -0.32561 | 0.890757 | 521.7907 | 483.172 | 1196.318 |
| 3 | 0.690515 | -0.88355 | 0.908125 | 255.7347 | 205.9643 | 799.6975 |
| 4 | 0.80946 | -1.10755 | 0.92254 | 143.6453 | 97.47956 | 575.041 |
| 5 | 0.848573 | -1.21417 | 0.931697 | 88.29314 | 50.37762 | 431.4229 |
| 6 | 0.868796 | -1.2576 | 0.941125 | 57.82264 | 27.45406 | 332.9563 |
| 7 | 0.882471 | -1.28619 | 0.950908 | 39.68145 | 15.86293 | 262.3381 |
| 8 | 0.879855 | -1.32017 | 0.946673 | 28.2265 | 9.522336 | 210.0651 |
| 9 | 0.882593 | -1.32911 | 0.948934 | 20.65653 | 5.896603 | 170.4399 |
| 10 | 0.892585 | -1.3232 | 0.961613 | 15.46964 | 3.739179 | 139.8316 |
| 12 | 0.881079 | -1.35208 | 0.957642 | 9.163137 | 1.622663 | 96.73403 |
| 14 | 0.857041 | -1.37715 | 0.941071 | 5.742792 | 0.767653 | 68.93747 |
| 16 | 0.868514 | -1.36071 | 0.957494 | 3.758947 | 0.382054 | 50.31659 |
| 18 | 0.867117 | -1.36502 | 0.957066 | 2.547759 | 0.196687 | 37.4583 |
| 20 | 0.875853 | -1.35964 | 0.966365 | 1.777543 | 0.101019 | 28.3541 |

| Table S2: the number of genes in the 17 modules | |
| --- | --- |
| moduleColors Freq | |
| black | 234 |
| green | 377 |
| magenta | 216 |
| red | 303 |
| blue | 633 |
| greenyellow | 156 |
| midnightblue | 54 |
| salmon | 125 |
| brown | 633 |
| grey | 261 |
| pink | 228 |
| tan | 140 |
| cyan | 100 |
| lightcyan | 52 |
| purple | 184 |
| turquoise | 1239 |
| yellow | 479 |

| Table S3 GO and KEGG pathway enrichment analysis of red module | | | | | |
| --- | --- | --- | --- | --- | --- |
| Category | Term | | Count | % | P-value |
| BP | GO:0019731~antibacterial humoral response | | 10 | 3.472222 | 9.48E-09 |
| BP | GO:0002227~innate immune response in mucosa | | 8 | 2.777778 | 4.19E-08 |
| BP | GO:0006334~nucleosome assembly | | 10 | 3.472222 | 5.13E-05 |
| BP | GO:0050830~defense response to Gram-positive bacterium | | 8 | 2.777778 | 0.000208 |
| BP | GO:0030218~erythrocyte differentiation | | 6 | 2.083333 | 0.000245 |
| BP | GO:0001878~response to yeast | | 4 | 1.388889 | 0.000733 |
| BP | GO:0015671~oxygen transport | | 4 | 1.388889 | 0.001141 |
| BP | GO:0044130~negative regulation of growth of symbiont in host | | 4 | 1.388889 | 0.00139 |
| BP | GO:0006952~defense response | | 6 | 2.083333 | 0.00264 |
| BP | GO:0045766~positive regulation of angiogenesis | | 7 | 2.430556 | 0.006057 |
| CC | GO:0070062~extracellular exosome | | 70 | 24.30556 | 8.43E-07 |
| CC | GO:0000788~nuclear nucleosome | 8 | | 2.777778 | 2.26E-06 |
| CC | GO:0005833~hemoglobin complex | 5 | | 1.736111 | 1.65E-05 |
| CC | GO:0005615~extracellular space | 39 | | 13.54167 | 2.24E-05 |
| CC | GO:0042581~specific granule | 5 | | 1.736111 | 2.35E-05 |
| CC | GO:0000786~nucleosome | 9 | | 3.125 | 4.91E-05 |
| CC | GO:0030863~cortical cytoskeleton | 5 | | 1.736111 | 0.000218 |
| CC | GO:0005856~cytoskeleton | 15 | | 5.208333 | 0.000717 |
| CC | GO:0005887~integral component of plasma membrane | 33 | | 11.45833 | 0.004279 |
| CC | GO:0042582~azurophil granule | 3 | | 1.041667 | 0.009721 |
| MF | GO:0046982~protein heterodimerization activity | 17 | | 5.902778 | 0.000728 |
| MF | GO:0005344~oxygen transporter activity | 4 | | 1.388889 | 0.000834 |
| MF | GO:0030492~hemoglobin binding | 3 | | 1.041667 | 0.001108 |
| MF | GO:0019825~oxygen binding | 5 | | 1.736111 | 0.003903 |
| MF | GO:0005506~iron ion binding | 8 | | 2.777778 | 0.00527 |
| MF | GO:0001047~core promoter binding | 5 | | 1.736111 | 0.011607 |
| MF | GO:0002020~protease binding | 6 | | 2.083333 | 0.012805 |
| MF | GO:0005521~lamin binding | 3 | | 1.041667 | 0.017553 |
| MF | GO:0043539~protein serine/threonine kinase activator activity | 3 | | 1.041667 | 0.024897 |
| MF | GO:0045296~cadherin binding | 3 | | 1.041667 | 0.039369 |
| MF | GO:0020037~heme binding | 6 | | 2.083333 | 0.040855 |
| MF | GO:0051537~2 iron, 2 sulfur cluster binding | 3 | | 1.041667 | 0.045859 |
| KEGG | hsa05322: Systemic lupus erythematosus | 11 | | 3.819444 | 3.1E-05 |
| KEGG | hsa05144: Malaria | 5 | | 1.736111 | 0.0062 |
| KEGG | hsa05034: Alcoholism | 8 | | 2.777778 | 0.017379 |
| KEGG | hsa05203: Viral carcinogenesis | 8 | | 2.777778 | 0.035165 |
| KEGG | hsa04640: Hematopoietic cell lineage | 5 | | 1.736111 | 0.042251 |
| Abbreviations: GO, gene Ontology; BP, biological process; CC, cellular component; MF, molecular function; KEGG, Kyoto encyclopedia of genes and genomes. | | | | | |

| Table S4 GO and KEGG pathway enrichment analysis of greenyellow module | | | | |
| --- | --- | --- | --- | --- |
| Category | Term | Count | % | P-value |
| BP | GO:0050776~regulation of immune response | 12 | 0.066379 | 5.95E-08 |
| BP | GO:0006955~immune response | 16 | 0.088505 | 3.25E-07 |
| BP | GO:0006968~cellular defense response | 7 | 0.038721 | 5.21E-06 |
| BP | GO:0006954~inflammatory response | 10 | 0.055316 | 0.0016 |
| BP | GO:0002548~monocyte chemotaxis | 4 | 0.022126 | 0.003335 |
| BP | GO:0048568~embryonic organ development | 3 | 0.016595 | 0.006421 |
| BP | GO:0042733~embryonic digit morphogenesis | 4 | 0.022126 | 0.007491 |
| BP | GO:0032332~positive regulation of chondrocyte differentiation | 3 | 0.016595 | 0.007999 |
| BP | GO:0045879~negative regulation of smoothened signaling pathway | 3 | 0.016595 | 0.007999 |
| BP | GO:0008045~motor neuron axon guidance | 3 | 0.016595 | 0.007999 |
| CC | GO:0005886~plasma membrane | 55 | 0.304237 | 4.18E-06 |
| CC | GO:0005887~integral component of plasma membrane | 24 | 0.132758 | 0.00028 |
| CC | GO:0016021~integral component of membrane | 56 | 0.309769 | 0.001255 |
| CC | GO:0009897~external side of plasma membrane | 7 | 0.038721 | 0.005124 |
| MF | GO:0032393~MHC class I receptor activity | 4 | 0.022126 | 7.61E-06 |
| MF | GO:0004888~transmembrane signaling receptor activity | 9 | 0.049784 | 0.000183 |
| MF | GO:0004872~receptor activity | 9 | 0.049784 | 0.000201 |
| MF | GO:0008013~beta-catenin binding | 5 | 0.027658 | 0.003119 |
| MF | GO:0098531~transcription factor activity, direct ligand regulated sequence-specific DNA binding | 2 | 0.011063 | 0.014638 |
| MF | GO:0018636~phenanthrene 9,10-monooxygenase activity | 2 | 0.011063 | 0.029063 |
| MF | GO:0008142~oxysterol binding | 2 | 0.011063 | 0.036196 |
| KEGG | hsa04612: Antigen processing and presentation | 14 | 0.077442 | 1.62E-13 |
| KEGG | hsa04650: Natural killer cell mediated cytotoxicity | 15 | 0.082974 | 5.09E-12 |
| KEGG | hsa04060: Cytokine-cytokine receptor interaction | 10 | 0.055316 | 0.00047 |
| KEGG | hsa05321: Inflammatory bowel disease (IBD) | 5 | 0.027658 | 0.003189 |
| KEGG | hsa04340: Hedgehog signaling pathway | 3 | 0.016595 | 0.027275 |
| KEGG | hsa05332: Graft-versus-host disease | 3 | 0.016595 | 0.039553 |
| KEGG | hsa05330: Allograft rejection | 3 | 0.016595 | 0.048691 |
| Abbreviations: GO, gene Ontology; BP, biological process; CC, cellular component; MF, molecular function; KEGG, Kyoto encyclopedia of genes and genomes. | | | | |

TABLE S5 The hub genes in the red module.

| nodeName | adj.P.Val | P.Value | logFC | IMConn |
| --- | --- | --- | --- | --- |
| EPB42 | 3.57E-19 | 4.58E-22 | 3.519417 | 51.38283 |
| ABCC13 | 1.55E-18 | 2.63E-21 | 2.705666 | 51.28703 |
| KLF1 | 4.74E-19 | 6.24E-22 | 2.707784 | 51.00958 |
| TRIM58 | 7.23E-20 | 7E-23 | 3.071353 | 49.89288 |
| AHSP | 3.79E-21 | 2.69E-24 | 3.71548 | 49.77129 |
| SNCA | 1.1E-15 | 4.02E-18 | 2.926766 | 49.63642 |
| OSBP2 | 2.43E-20 | 2.13E-23 | 2.391383 | 49.57803 |
| SELENBP1 | 2.91E-19 | 3.62E-22 | 3.368002 | 49.25572 |
| DCAF12 | 3.54E-18 | 6.42E-21 | 1.494508 | 48.86795 |
| FAM210B | 2.66E-15 | 1.07E-17 | 2.163564 | 48.64206 |
| DMTN | 3.75E-16 | 1.25E-18 | 1.996333 | 48.16349 |
| GMPR | 1.11E-22 | 4.28E-26 | 2.86788 | 48.06731 |
| ANK1 | 1.77E-19 | 1.84E-22 | 2.327358 | 46.42753 |
| CA1 | 1.37E-20 | 1.15E-23 | 3.894928 | 46.28165 |
| SLC4A1 | 6.89E-19 | 1.01E-21 | 3.763306 | 46.23996 |
| YOD1 | 7.02E-18 | 1.41E-20 | 1.847313 | 45.75891 |
| GLRX5 | 2.1E-16 | 6.72E-19 | 1.475626 | 45.56773 |
| HBQ1 | 1.08E-23 | 2.96E-27 | 2.167325 | 45.18133 |
| ADIPOR1 | 6.32E-19 | 8.91E-22 | 1.45454 | 45.04866 |
| HBM | 1.46E-22 | 5.88E-26 | 3.165674 | 45.03217 |
| BPGM | 1.94E-19 | 2.1E-22 | 2.11662 | 44.98007 |
| ALAS2 | 2.04E-22 | 9.31E-26 | 4.45908 | 44.77448 |
| GYPA | 6.78E-19 | 9.69E-22 | 3.724885 | 44.51569 |
| IGF2BP2 | 8.42E-17 | 2.34E-19 | 1.939746 | 43.104 |
| TSPAN5 | 1.52E-15 | 5.76E-18 | 1.85399 | 42.8392 |
| LOC102724387 | 4.09E-22 | 2.17E-25 | 2.952147 | 42.73936 |
| GYPB | 5.88E-18 | 1.15E-20 | 3.721733 | 42.50459 |
| TNS1 | 5.07E-17 | 1.31E-19 | 2.679436 | 40.90252 |
| TRIM10 | 1.88E-18 | 3.26E-21 | 3.14703 | 40.7499 |
| NPRL3 | 1.22E-14 | 5.72E-17 | 1.51048 | 40.58929 |

TABLE S6 The hub genes in the greenyellow module.

| nodeName | adj.P.Val | P.Value | logFC | IMConn |
| --- | --- | --- | --- | --- |
| AGAP1 | 9.4E-13 | 7.18E-15 | -0.82878 | 6.551727 |
| C1orf21 | 6.64E-05 | 6.32E-06 | -0.52041 | 7.464688 |
| CD160 | 1.09E-09 | 1.84E-11 | -1.15041 | 7.472294 |
| DLG5 | 2.63E-06 | 1.41E-07 | -0.75479 | 6.47601 |
| ENPP5 | 6.4E-08 | 1.94E-09 | -0.67377 | 7.320619 |
| EOMES | 6.51E-06 | 4.12E-07 | -0.49503 | 8.594299 |
| FGFBP2 | 2.71E-07 | 1.02E-08 | -0.8326 | 8.598293 |
| GPR56 | 9.42E-06 | 6.36E-07 | -0.78804 | 7.669137 |
| GZMA | 1.56E-06 | 7.77E-08 | -0.53609 | 7.162492 |
| HOPX | 1.16E-09 | 1.97E-11 | -0.83951 | 8.109679 |
| IL2RB | 6.76E-07 | 2.94E-08 | -0.64581 | 8.358424 |
| KIR2DL1 | 0.011515 | 0.003187 | -0.50836 | 6.765851 |
| KIR2DL2 | 0.005131 | 0.001183 | -0.53016 | 8.11722 |
| KIR2DL3 | 0.032322 | 0.01134 | -0.32231 | 6.976707 |
| KIR3DL1 | 0.002637 | 0.000524 | -0.46758 | 6.475435 |
| KIR3DL3 | 0.017455 | 0.00533 | -0.47188 | 7.00745 |
| KLRB1 | 3.38E-16 | 1.12E-18 | -0.99617 | 6.649343 |
| KLRF1 | 1.56E-11 | 1.66E-13 | -1.05756 | 10.532 |
| MYBL1 | 8.61E-13 | 6.53E-15 | -0.88201 | 10.03416 |
| NCALD | 2.19E-10 | 3.08E-12 | -0.5971 | 7.22221 |
| PDGFD | 3.17E-09 | 6.22E-11 | -0.90577 | 8.88934 |
| PDZD4 | 1.63E-08 | 4.09E-10 | -0.83621 | 8.791942 |
| PHLDB2 | 5.3E-12 | 5.18E-14 | -0.97666 | 8.633386 |
| PPP2R2B | 3.35E-13 | 2.25E-15 | -1.06892 | 10.11099 |
| PRSS23 | 2.48E-13 | 1.62E-15 | -1.45232 | 10.91953 |
| PTGDR | 3.9E-10 | 5.91E-12 | -0.96261 | 8.421833 |
| S1PR5 | 1.36E-06 | 6.62E-08 | -0.84828 | 10.81499 |
| SH2D1B | 4.65E-07 | 1.91E-08 | -0.82587 | 6.689891 |
| TBX21 | 6.18E-07 | 2.65E-08 | -0.6927 | 9.785981 |
| TGFBR3 | 4.45E-06 | 2.63E-07 | -0.6065 | 8.407776 |

| Table S7 GO enrichment analysis of hub genes in red and greenyellow module | | | | | | |
| --- | --- | --- | --- | --- | --- | --- |
| Category | | Term | Count | % | P-value | Genes |
| Red module | | |  |  |  |  |
| BP | GO:0030218~erythrocyte differentiation | | 4 | 0.10543 | 3.13E-05 | ALAS2, AHSP, TRIM10, KLF1 |
| BP | GO:0020027~hemoglobin metabolic process | | 2 | 0.052715 | 0.009256 | EPB42, AHSP |
| BP | GO:0015671~oxygen transport | | 2 | 0.052715 | 0.022985 | HBM, HBQ1 |
| BP | GO:0048821~erythrocyte development | | 2 | 0.052715 | 0.022985 | DMTN, BPGM |
| BP | GO:0007010~cytoskeleton organization | | 3 | 0.079072 | 0.02554 | DMTN, ANK1, EPB42 |
| BP | GO:0015701~bicarbonate transport | | 2 | 0.052715 | 0.06599 | SLC4A1, CA1 |
| BP | GO:0030097~hemopoiesis | | 2 | 0.052715 | 0.087514 | GLRX5, AHSP |
| Greenyellow module | | | |  |  |  |
| BP | GO:0050776~regulation of immune response | | 8 | 27.58621 | 3.97E-09 | KLRB1, CD160, KIR2DL1, KLRF1, KIR2DL3, KIR2DL2, SH2D1B, KIR3DL1 |
| BP | GO:0006955~immune response | | 5 | 17.24138 | 0.00278 | GZMA, TGFBR3, KIR2DL1, KIR2DL3, KIR3DL1 |
| BP | GO:0001829~trophectodermal cell differentiation | | 2 | 6.896552 | 0.018428 | HOPX, EOMES |
| BP | GO:0043393~regulation of protein binding | | 2 | 6.896552 | 0.026823 | HOPX, TGFBR3 |
| BP | GO:0045664~regulation of neuron differentiation | | 2 | 6.896552 | 0.028216 | S1PR5, EOMES |
| BP | GO:0007166~cell surface receptor signaling pathway | | 3 | 10.34483 | 0.057858 | KLRB1, CD160, KLRF1 |
| Abbreviations: GO, gene Ontology; BP, biological process. | | | | | | |
