# Supplementary material for: Application of Weighted Gene Coexpression Network Analysis to Identify Key Modules and Hub Genes in Systemic Juvenile Idiopathic Arthritis
Source: Biomed Res Int. 2021 Aug 13;2021:9957569. doi: 10.1155/2021/9957569 (PMC8382540; doi:10.1155/2021/9957569)
Supplement: Supplementary Materials — Supplementary 1 Figure S1: sample clustering to detect outliers. Figure S2: analysis of network topology for various soft-thresholding powers. Figure S3: construction of the consensus WGCNA. Figure S4: bar plot of mean gene significance across modules. Figure S5A-S5C: interactions between module hub genes and genome-wide-associated genes. Table S1: analysis of network topology for various soft-thresholding powers. Table S2: the number of genes in each module. Table S3: the GO and KEGG pathway enrichment analysis of the red module. Table S4: the GO and KEGG pathway enrichment analysis of the green-yellow module. Table S5: the hub genes in the red module. Table S6: the hub genes in the green-yellow module. Table S7: the GO enrichment analysis of hub genes in red and green-yellow module. Supplementary 2 WGCNA codes. [file 9957569.f1.zip › 9957569.f1/Supplementary2_codes_updated.docx]

Supplementary Material 2

WGCNA Codes

##################################################################

#Data collection and data quality control

#1) Affymetrix

library(affy)

library(hgu133plus2cdf)

#1.1) RMA normalization and preprocess the raw data

##the raw data and group

SmpGrp <- read.csv('data/sample_group.csv', header = T)

rownames(SmpGrp) <- SmpGrp[ ,1]

SmpGrp <- SmpGrp[ ,2:3]

##rma

##Background correction

##normalization

##Expression calculation

Data <- ReadAffy(filenames = paste(rownames(SmpGrp), '.CEL', sep = ''),

celfile.path = "./data/RAW")

eset <- rma(Data)

dataExp <- exprs(eset)

#1.2) KNN supplement missing values

library(impute)

dataknn <- impute.knn(dataExp, k=10, rowmax = 0.5,

colmax = 0.8, maxp = 1500,

rng.seed = 362436069)

dataExp2 <- dataknn$data

#1.3) sva-ComBat-batch effect)

library(sva)

batch <- SmpGrp[ ,c('batch')]

batch

#

ComBatmodel <- model.matrix(~group, data = SmpGrp)

#ComBat

dataCombat <- ComBat(dat = dataExp2,batch = batch,

mod = ComBatmodel, par.prior = T, prior.plot = T)

ExpMatrix <- dataCombat

#renew page

colnames(ExpMatrix) <- rownames(SmpGrp)

#1.4) Check the elimination of batch effects,load batch_review.R

#2) Visualize data distribution and generate expression matrix tables

#2.1) Visualize data distribution

library(ggplot2)

library(reshape2)

ExpMatrix_L <- melt(ExpMatrix)

colnames(ExpMatrix_L) <- c('probe', 'sample', 'value')

group <- factor(SmpGrp$group, levels = c("sJIA", "Health"))

ExpMatrix_L$group <- rep(group, each = nrow(ExpMatrix))

head(ExpMatrix_L)

##boxplot

ggplot(ExpMatrix_L,aes(x=sample,y=value,fill=group)) +

geom_boxplot() +

stat_summary(fun.y = "mean",geom = "point",shape = 1, size = 1.5) +

theme_set(theme_set(theme_bw(base_size =8 ))) +

theme(text = element_text(face = 'bold'),

axis.text.x = element_text(angle = 90, vjust = 0.5, hjust = 1),

axis.title = element_blank())

##PCA

library(ggfortify)

pca <- as.data.frame(t(ExpMatrix))

pca$group <- group

autoplot(prcomp(pca[ ,1:(ncol(pca)-1)] ), data = pca, colour = 'group')+

theme_set(theme_set(theme_bw(base_size=8)))

##heatmap

library(pheatmap)

choose_gene <- names(tail(sort(apply(ExpMatrix, 1, mad)), 100))

choose_matrix <- ExpMatrix[choose_gene, ]

choose_matrix <- t(scale(t(choose_matrix)))

pheatmap(choose_matrix, annotation_col = SmpGrp, fontsize = 8)

#display_numbers = TRUE, number_format = "%.2f", number_color="purple" #50*100

#2.2) generate expression matrix tables

write.csv(ExpMatrix, file="ExpMatrix.csv", quote = F, na = "", row.names = T)

save(group,ExpMatrix,SmpGrp,file = "step1output.Rdata")

###########################################################################

#group_list,probe annotation

rm(list = ls())

load(file = "step1output.Rdata")

dataExp <- ExpMatrix

#probe annotation

library(idmap2)

ids=get_soft_IDs('gpl570')

ID <- rownames(dataExp)

dataExp1=data.frame(dataExp,ID)

library(dplyr)

dataExp2 <- left_join(dataExp1,ids,by="ID")

####Merge probe

head(dataExp2)

##### delete"///"

dataExp20 <- filter(dataExp2,!grepl("///",symbol))

#Merge probe based on the max

dataExp22 <- aggregate(x = dataExp20[,1:127],

by = list(dataExp20$symbol),

FUN = max)

dataExp3 <- dataExp22

rownames(dataExp3) <-dataExp3[,1]

dataExp4 <-dataExp3[ ,2:128]

######################################################

#WGCNA

#step1:data preparation

library(WGCNA)

##Select the top 25% of genes with the largest variance as the input data set for subsequent WGCNA

m.vars=apply(dataExp4,1,mad)

WGCNA_matrix1=dataExp4[which(m.vars>quantile(m.vars, probs = seq(0, 1, 0.25))[4]),]

dim(WGCNA_matrix1)

##########################

#Test for differences between samples

datExpr=as.data.frame(t(WGCNA_matrix1));

gsg = goodSamplesGenes(datExpr, verbose = 3);

gsg$allOK

sampleTree = hclust(dist(datExpr), method = "average")

plot(sampleTree, main = "Sample clustering to detect outliers"

, sub="", xlab="")

clust = cutreeStatic(sampleTree, cutHeight = 1000, minSize = 10)

table(clust)

#clust

#0 1

#2 98

keepSamples = (clust==1)

#Get clinical information

datTraits <- SmpGrp

datExpr = datExpr[keepSamples, ]

datTraits <- SmpGrp[keepSamples, ]

nGenes = ncol(datExpr)

nSamples = nrow(datExpr)

###################################

####end

save(datExpr,datTraits,file = 'GSE100648-wgcna-input.RData')

rm(list = ls())

load(file = 'GSE100648-wgcna-input.RData')

#################################################################################

#

dev.off()

#

nGenes = ncol(datExpr)

nSamples = nrow(datExpr)

#'Sample clustering'

datExpr_tree<-hclust(dist(datExpr), method = 'average')

par(mar = c(0,5,2,0))

plot(datExpr_tree, main = 'Sample clustering', sub='', xlab='', cex.lab = 2,

cex.axis = 1, cex.main = 1,cex.lab=1)

sample_colors <- numbers2colors(as.numeric(factor(datTraits$group)),

colors = c('blue','red'),signed = FALSE)

par(mar = c(1,4,3,1),cex=0.8)

plotDendroAndColors(datExpr_tree, sample_colors,

groupLabels = colnames(sample),

cex.dendroLabels = 0.8,

marAll = c(1, 4, 3, 1),

cex.rowText = 0.01,

main = 'Sample dendrogram and trait heatmap')

################################################################################

###################################################################################

#step2:Determine the best beta value

library(WGCNA)

#soft thresholding power”beta：

powers = c(c(1:10), seq(from = 12, to=20, by=2))

# Call the network topology analysis function

sft = pickSoftThreshold(datExpr, powerVector = powers, verbose = 5)

#

# Plot the results:

##sizeGrWindow(9, 5)

par(mfrow = c(1,2));

cex1 = 0.9;

# Scale-free topology fit index as a function of the soft-thresholding power

plot(sft$fitIndices[,1], -sign(sft$fitIndices[,3])*sft$fitIndices[,2],

xlab='Soft Threshold (power)',ylab='Scale Free Topology Model Fit,signed R^2',type='n',

main = paste('Scale independence'));

text(sft$fitIndices[,1], -sign(sft$fitIndices[,3])*sft$fitIndices[,2],

labels=powers,cex=cex1,col='red');

# this line corresponds to using an R^2 cut-off of h

abline(h=0.90,col='red')

# Mean connectivity as a function of the soft-thresholding power

plot(sft$fitIndices[,1], sft$fitIndices[,5],

xlab='Soft Threshold (power)',ylab='Mean Connectivity', type='n',

main = paste('Mean connectivity'))

text(sft$fitIndices[,1], sft$fitIndices[,5], labels=powers, cex=cex1,col='red')

#pickSoftThreshold

#prefect beta:sft$powerEstimate

beta <- sft$powerEstimate

save(datExpr,datTraits,sft,file = 'GSE100648-wgcna-input2.RData')

rm(list = ls())

load(file = 'GSE100648-wgcna-input2.RData')

################################################################################

#step3：One-step construction of co-expression matrix

#

net = blockwiseModules(

datExpr,

power = sft$powerEstimate,

maxBlockSize = 6000,#Set a maximum number, not too small;

TOMType = 'unsigned', minModuleSize = 40,#Smallest node;

reassignThreshold = 0, mergeCutHeight = 0.25,

numericLabels = TRUE, pamRespectsDendro = FALSE,

saveTOMs = F,

verbose = 3

)

table(net$colors)

save(datExpr,datTraits,sft,net,file = 'GSE100648-wgcna-input3.RData')

rm(list = ls())

load(file = 'GSE100648-wgcna-input3.RData')

#################################################################################

#step4: Module visualization

dev.off()

# Convert labels to colors for plotting

mergedColors = labels2colors(net$colors)

table(mergedColors)

# Plot the dendrogram and the module colors underneath

plotDendroAndColors(net$dendrograms[[1]], mergedColors[net$blockGenes[[1]]],

'Module colors',

dendroLabels = FALSE, hang = 0.03,

addGuide = TRUE, guideHang = 0.05)

## assign all of the gene to their corresponding module

## hclust for the genes.

#################################################################################

#step5:Relationship between modules and clinical traits

## step 5 Relationship between modules and clinical traits

table(datTraits$group)

if(T){

nGenes = ncol(datExpr)

nSamples = nrow(datExpr)

design=model.matrix(~0+ datTraits$group)

colnames(design)=levels(datTraits$group)

moduleColors <- labels2colors(net$colors)

# Recalculate MEs with color labels

MEs0 = moduleEigengenes(datExpr, moduleColors)$eigengenes

MEs = orderMEs(MEs0); ##

moduleTraitCor = cor(MEs, design , use = 'p');

moduleTraitPvalue = corPvalueStudent(moduleTraitCor, nSamples)

sizeGrWindow(10,6)

# Will display correlations and their p-values

textMatrix = paste(signif(moduleTraitCor, 2), '\n(',

signif(moduleTraitPvalue, 1), ')', sep = '');

dim(textMatrix) = dim(moduleTraitCor)

png('step5-Module-trait-relationships.png',width = 800,height = 1200,res = 120)

par(mar = c(6, 8.5, 3, 3));

# Display the correlation values within a heatmap plot

design=model.matrix(~0+ datTraits$group)

labeledHeatmap(Matrix = moduleTraitCor,

xLabels = colnames(design),

yLabels = names(MEs),

ySymbols = names(MEs),

colorLabels = FALSE,

colors = blueWhiteRed(50),

textMatrix = textMatrix,

setStdMargins = FALSE,

cex.text = 0.5,

zlim = c(-1,1),

main = paste('Module-trait relationships'))

dev.off()

#

Health = as.data.frame(design[,1]);

names(Health) = 'Health'

y=Health

GS1=as.numeric(cor(y,datExpr, use='p'))

GeneSignificance=abs(GS1)

# Next module significance is defined as average gene significance.

ModuleSignificance=tapply(GeneSignificance,

moduleColors, mean, na.rm=T)

sizeGrWindow(8,7)

par(mfrow = c(1,1))

#

plotModuleSignificance(GeneSignificance,moduleColors)

}

##############################################################################

#step6:Specific gene analysis of modules of interest traits

#First calculate the correlation matrix between the module and the gene

# names (colors) of the modules

modNames = substring(names(MEs), 3)

geneModuleMembership = as.data.frame(cor(datExpr, MEs, use = 'p'));

## Calculate the Pearson correlation coefficient matrix of each module and gene

## MEs Is the value of each module in each sample

## datExpr Is the expression level of each gene in each sample

MMPvalue = as.data.frame(corPvalueStudent(as.matrix(geneModuleMembership), nSamples));

names(geneModuleMembership) = paste('MM', modNames, sep='');

names(MMPvalue) = paste('p.MM', modNames, sep='');

#Recalculate the correlation matrix between traits and genes

## Only continuous traits can only be calculated

## Here, whether the variable belongs to the sJIA phenotype is numericalized with 0,1.

sJIA = as.data.frame(design[,2]);

names(sJIA ) = 'sJIA'

geneTraitSignificance = as.data.frame(cor(datExpr, sJIA , use = 'p'));

GSPvalue = as.data.frame(corPvalueStudent(as.matrix(geneTraitSignificance), nSamples));

names(geneTraitSignificance) = paste('GS.', names(sJIA ), sep='');

names(GSPvalue) = paste('p.GS.', names(sJIA ), sep='');

# Finally, combine the two correlation matrices and specify the module of interest for analysis

#### Select greenyellow module;

#### Study the red module in the same way

module = 'greenyellow'

column = match(module, modNames);

moduleGenes = moduleColors==module;

sizeGrWindow(7, 7);

par(mfrow = c(1,1));

verboseScatterplot(abs(geneModuleMembership[moduleGenes, column]),

abs(geneTraitSignificance[moduleGenes, 1]),

xlab = paste('Module Membership in', module, 'module'),

ylab = 'Gene significance for H',

main = paste('Module membership vs. gene significance\n'),

cex.main = 1.2, cex.lab = 1.2, cex.axis = 1.2, col = module)

#Comparison of specified genes in modules and traits

#It can be seen that these genes are not only highly related to their corresponding modules;

#And it is highly correlated with its corresponding trait, further illustrating that the gene is worth exploring in depth.

##############################################################################

#step7:Visualization of the network

#First draw a heat map for all genes

# Mainly visualize the TOM matrix, WGCNA's standard map

# Then visualize the correlation of different modules Heatmap

# Hierarchical clustering diagram of different modules

# There is also module diagnosis, mainly intramodular connectivity

nGenes = ncol(datExpr)

nSamples = nrow(datExpr)

geneTree = net$dendrograms[[1]];

TOM = TOMsimilarityFromExpr(datExpr, power = sft$powerEstimate);

dissTOM = 1-TOM;

plotTOM = dissTOM^7;

diag(plotTOM) = NA;

#Select = 1000 or all genes(slow)

TOMplot(plotTOM, geneTree, moduleColors, main = 'Network heatmap plot, all genes')

nSelect = 1000

# For reproducibility, we set the random seed

set.seed(10);

select = sample(nGenes, size = nSelect);

selectTOM = dissTOM[select, select];

# There’s no simple way of restricting a clustering tree to a subset of genes, so we must re-cluster.

selectTree = hclust(as.dist(selectTOM), method = 'average')

selectColors = moduleColors[select];

# Open a graphical window

sizeGrWindow(9,9)

# Taking the dissimilarity to a power, say 10, makes the plot more informative by effectively changing

# the color palette; setting the diagonal to NA also improves the clarity of the plot

plotDiss = selectTOM^7;

diag(plotDiss) = NA;

png('step7-Network-heatmap.png',width = 800,height = 600)

TOMplot(plotDiss, selectTree, selectColors, main = 'Network heatmap plot, selected genes')

dev.off()

#The relationship between modules and traits

# Recalculate module eigengenes

MEs = moduleEigengenes(datExpr, moduleColors)$eigengenes

## Here, whether the variable 0,1 belongs to the sJIA phenotype is numericalized

sJIA = as.data.frame(design[,2]);

names(sJIA ) = 'sJIA '

# Add the weight to existing module eigengenes

MET = orderMEs(cbind(MEs, sJIA ))

# Plot the relationships among the eigengenes and the trait

sizeGrWindow(5,7.5);

par(cex = 0.9)

png('step7-Eigengene-dendrogram.png',width = 800,height = 600)

plotEigengeneNetworks(MET, '', marDendro = c(0,4,1,2), marHeatmap = c(3,4,1,2), cex.lab = 0.8, xLabelsAngle

= 90)

dev.off()

# Plot the dendrogram

sizeGrWindow(6,6);

par(cex = 1.0)

## module Eigengene-dendrogram-hclust

png('step7-Eigengene-dendrogram-hclust.png',width = 800,height = 600)

plotEigengeneNetworks(MET, 'Eigengene dendrogram', marDendro = c(0,4,2,0),

plotHeatmaps = FALSE)

dev.off()

# Plot the heatmap matrix (note: this plot will overwrite the dendrogram plot)

par(cex = 1.0)

## module Eigengene-adjacency-heatmap

png('step7-Eigengene-adjacency-heatmap.png',width = 800,height = 600)

plotEigengeneNetworks(MET, 'Eigengene adjacency heatmap', marHeatmap = c(3,4,2,2),

plotDendrograms = FALSE, xLabelsAngle = 90)

dev.off()

###############################################################################

#step8:Extract the gene name of the specified module

## step 8

# Mainly concerned about the genes inside a specific module

#Care about sJIA's red and greenyellow module

# HERE Extract the gene name of the greenyellow module

# the same way to study the red module

if(T){

# Select module

module = 'greenyellow';

# Select module probes

probes = colnames(datExpr) ## probe:gene

inModule = (moduleColors==module);

modProbes = probes[inModule];

head(modProbes)

# heatmap by WGCNA

which.module='greenyellow';

dat=datExpr[,moduleColors==which.module ]

plotMat(t(scale(dat)),nrgcols=30,rlabels=T,

clabels=T,rcols=which.module,

title=which.module )

datExpr[1:4,1:4]

dat=t(datExpr[,moduleColors==which.module ] )

library(pheatmap)

pheatmap(dat ,show_colnames =F,show_rownames = F)

n=t(scale(t(log(dat+1)))) #

n[n>2]=2

n[n< -2]= -2

n[1:4,1:4]

pheatmap(n,show_colnames =F,show_rownames = F)

group_list=datTraits$group

ac=data.frame(g=group_list)

rownames(ac)=colnames(n)

pheatmap(n,show_colnames =F,show_rownames = F,

annotation_col=ac )

}

#################################################################################

#Step9: Module export

#The direct interaction information of the main module de genes can be exported to cytoscape, VisANT and other network visualization software.

# Recalculate topological overlap

#

TOM = TOMsimilarityFromExpr(datExpr, power = sft$powerEstimate);

# Select module

#### Study the red module in the same way

module = 'greenyellow';

# Select module probes

probes = colnames(datExpr) ##

inModule = (moduleColors==module);

modProbes = probes[inModule];

##

# Select the corresponding Topological Overlap

modTOM = TOM[inModule, inModule];

dimnames(modTOM) = list(modProbes, modProbes)

## Gene relationship matrix corresponding to the module

#to cytoscape

cyt = exportNetworkToCytoscape(

modTOM,

edgeFile = paste('CytoscapeInput-edges-', paste(module, collapse='-'), '.txt', sep=''),

nodeFile = paste('CytoscapeInput-nodes-', paste(module, collapse='-'), '.txt', sep=''),

weighted = TRUE,

threshold = 0.02,#

nodeNames = modProbes,

nodeAttr = moduleColors[inModule]

);

#selest the top 30

nTop = 30;

IMConn = softConnectivity(datExpr[, modProbes]);

top = (rank(-IMConn) <= nTop)

filterred <- modTOM[top, top]

#topgene30

Topgene <- data.frame(modProbes,IMConn)[top,]
